# The relationship between linguistic and non-linguistic cognitive control skills in bilingual children from low socio-economic backgrounds

**DOI:** 10.3389/fpsyg.2014.01098

**Published:** 2014-09-26

**Authors:** Milijana Buac, Margarita Kaushanskaya

**Affiliations:** Communication Sciences and Disorders, Waisman Center, University of Wisconsin–MadisonMadison, WI, USA

**Keywords:** bilingualism, SES, syntactic processing, semantics, cognitive control

## Abstract

The present study examined whether linguistic cognitive control skills were related to non-linguistic cognitive control skills in monolingual children (Study 1) and in bilingual children from low socio-economic status (SES) backgrounds (Study 2). Linguistic inhibitory control was measured using a grammaticality judgment (GJ) task in which children judged the grammaticality of sentences while ignoring their meaning. Non-linguistic inhibitory control was measured using a flanker task. Study 1, in which we tested monolingual English-speaking children, revealed that better inhibitory control skills, as indexed by the performance on the flanker task, were associated with improved performance on the GJ task. Study 2, in which we tested bilingual English-Spanish speaking children from low SES backgrounds, revealed that better non-linguistic inhibitory control skills did *not* yield better performance on the GJ task. Together, these findings point to a role of domain-general attention mechanisms in language performance in typically developing monolingual children, but not in bilingual children from low SES. Present results suggest that the relationship between linguistic and domain-general cognitive-control abilities is instantiated differently in bilingual vs. monolingual children, and that language-EF interactions are sensitive to language status and SES.

## INTRODUCTION

A large body of research suggests that bilingualism may positively impact cognitive control mechanisms (e.g., Bialystok, 1999; Bialystok et al., 2004; Bialystok and Martin, 2004; Kroll et al., 2008; Kovács and Mehler, 2009) and executive functions (EF) in general. Executive functions refer to cognitive processes that aid in controlling and monitoring goal-directed behavior. They include the ability to inhibit irrelevant information and/or responses, the ability to shift between tasks or mental schemas, and the ability to update information in working memory (Miyake et al., 2000). Bilingual advantages have been observed in populations spanning a wide age range, from infancy to old age (e.g., Bialystok et al., 2004, 2005, 2006; Costa et al., 2008; Kovács and Mehler, 2009; Bialystok, 2010), and on a wide array of tasks requiring conflict resolution (e.g., Flanker: Carlson and Meltzoff, 2008; Costa et al., 2008; Simon: Bialystok, 2006; Martin-Rhee and Bialystok, 2008; Stroop: Bialystok et al., 2008). In such tasks, congruent, incongruent, and neutral stimuli are presented, where incongruent trials require inhibition of irrelevant information while attending to task-relevant information. Typically, when bilingual advantages are observed, they are observed on the incongruent trials that require increased cognitive control (e.g., Costa et al., 2008; Prior and MacWhinney, 2010), although recent studies have yielded overall bilingual advantages on cognitive control tasks, including the congruent trials (Costa et al., 2009).

The favored hypothesis for explaining these bilingual EF advantages is that bilinguals’ two languages are continuously activated and, therefore, bilingual speakers are required to continuously monitor their linguistic environment in order to inhibit the irrelevant language. Thus, executive control exercised at the linguistic level is theorized to generalize to the non-linguistic level (Bialystok, 2001) resulting in enhanced cognitive control skills. However, the relationship between the linguistic and the non-linguistic cognitive control systems is poorly understood, and very few studies have examined the link between the two control systems directly (but see Alario et al., 2012 for an exception). Furthermore, conflicting evidence with regard to bilingual EF advantages has been accumulating with an ever-growing number of studies yielding a lack of bilingual–monolingual differences on non-linguistic cognitive control measures (e.g., Morton and Harper, 2007; Hilchey and Klein, 2011; Paap and Greenberg, 2013; Paap, 2014). One factor that appears to contribute significantly to the ability to observe bilingual advantages on EF tasks is socio-economic status (SES; Morton and Harper, 2007). The goals of the present study were twofold. First, we aimed to contribute to the literature on the roots of bilingual EF advantages by exploring the relationship between linguistic and non-linguistic cognitive control skills in monolingual vs. bilingual children. Second, we aimed to contribute to the debate regarding the influences of SES to the development of linguistic and non-linguistic inhibitory control by exploring the relationship between them in two separate populations that represent the common demographic trends associated with monolingualism vs. bilingualism in the U.S.: a group of monolingual English-speaking children from middle SES backgrounds and a group of bilingual Spanish–English speaking children from low SES backgrounds.

### THE ROLE OF SES IN BILINGUAL EF PERFORMANCE

While reports of bilingual advantages on non-linguistic cognitive control tasks continue to appear regularly in the literature, a number of studies have also reported more complex results regarding bilingual performance on conflict resolution tasks. The nuanced nature of bilingual EF advantages appears to be conditioned both by the cognitive control tasks used (Hilchey and Klein, 2011) and by the socio-demographic characteristics of the populations tested (Morton and Harper, 2007). SES in particular, and the distinct SES niches occupied by bilingual vs. monolingual populations in the United States, has become the focus of much debate on the differences between bilinguals’ and monolinguals’ EF skills.

It is well documented that SES impacts children’s cognitive development (Liaw and Brooks-Gunn, 1994; Smith et al., 1997; Hoff, 2003; Mezzacappa, 2004; Ardila et al., 2005; Hughes and Ensor, 2005; Noble et al., 2005). A study by the National Institute of Child Health, and Human Development (NICHD) Early Child Care Research Network (2003) revealed that the quality of the child’s home environment was a significant predictor of children’s executive control and children’s ability to sustain attention. Similarly, Mezzacappa (2004) examined the impact of socio-demographic characteristics on children’s performance on the Attentional Network Test (ANT; Fan et al., 2002), and found that children from high SES backgrounds were more accurate and faster on the task compared to children from lower SES backgrounds. A more recent study by Kishiyama et al. (2009) examined prefrontal cortex functioning using electrophysiological data in children from low SES and children from higher SES. Kishiyama et al. (2009) found that measures of attention yielded reduced activation of the prefrontal cortex in children from lower SES when compared to children from higher SES. Therefore, neurophysiological evidence is consistent with behavioral studies indicating that children from lower SES environments are at a disadvantage on measures of executive functions.

One main criticism of research examining differences between monolingual and bilingual cognitive control mechanisms has been the lack of control of factors such as SES. A recent series of commentaries between Bialystok (2009) and Morton and Harper (2009) specifically focused on this matter. While Morton and Harper (2009) maintained that SES may have been confounded with bilingualism in prior studies that have observed bilingual EF advantages (and in fact observed no differences between the groups of monolingual and bilingual children matched on SES), Bialystok (2009) argued that prior studies on bilingual advantages conducted in her laboratory had taken SES into consideration by recruiting children from the same schools and neighborhoods (thus implying similar SES backgrounds).

Several recent studies have explicitly considered the impact of SES on bilingual children’s EF performance, and found bilingual advantages (Carlson and Meltzoff, 2008; de Abreu et al., 2012; Calvo and Bialystok, 2014). For example, a recent study by Calvo and Bialystok (2014) demonstrated that bilingualism and SES have a significant *but independent* impact on bilingual children’s performance on tasks of executive functions. Calvo and Bialystok (2014) recruited two groups of monolingual and bilingual children: children from working class families and children from middle class families. Results revealed that children from middle class families outperformed children from working class families on measures of vocabulary and EF, regardless of language background. However, the impact of bilingualism and SES did not interact; bilingual children obtained lower English vocabulary scores compared to their monolingual peers (regardless of which SES group they were in), but also outperformed monolingual children on EF tasks. The authors interpreted these results to mean that bilingualism decelerates bilinguals’ vocabulary development while at the same time accelerating EF skills, independent of SES.

One other study attempted to control for the impact of SES on bilinguals’ EF performance by comparing a group of low SES bilinguals to a group of monolingual peers also from a low SES background (de Abreu et al., 2012). Specifically, de Abreu et al. (2012) examined cognitive control skills in a group of low SES children from Portugal who immigrated to Luxemburg and were being raised as Portugal-Luxemburgish bilinguals, and compared them to their monolingual peers from low SES families who resided in Portugal. Results showed bilingual advantages in conflict resolution, suggesting that bilingualism in the context of a low SES environment can yield EF advantages.

A different approach to examining the effects of SES on bilingual EF skills was taken by Carlson and Meltzoff (2008), who carefully covaried factors that differentiated the bilingual and the monolingual children in their study, including SES. Carlson and Meltzoff (2008) administered a number of EF measures to monolingual English-speaking children, bilingual Spanish–English-speaking children, and native English-speaking children enrolled in Spanish–English dual immersion programs. Because analyses revealed significant between-group differences in age, SES, and verbal ability, Carlson and Meltzoff (2008) statistically controlled for those variables in all between-group comparisons. Results revealed a bilingual advantage where the bilingual group outperformed both the monolingual group and the dual-immersion group once differences in age, verbal ability, and SES were statistically controlled. However, no differences on EF measures were observed among the three groups when these factors were not covaried.

Thus, attempts to control for SES in prior literature on bilingual EF advantages appear to suggest that although SES plays an important role in EF development, bilingualism can contribute to EF skills independently and positively. However, one difficulty with interpreting the results of these prior studies on the interaction between SES and bilingualism in shaping EF skills is the problematic nature of the approaches taken to examine these interactions. With regard to de Abreu et al. (2012) approach, matching the levels of SES across monolingual and bilingual children did not eliminate the issue of comparing immigrant children living in one country to non-immigrant children living in another country. Immigration status may have significant repercussions for life experiences that can contribute to EF performance (such as schooling, for example), and can contaminate bilingual/monolingual comparisons when the two populations are drawn from different countries (see Paap, 2014 for a similar interpretation). With regard to Carlson and Meltzoff (2008) approach, statistically covarying SES in the analyses of EF skills is problematic because SES is strongly linked to the dependent variable in such analyses. This can result in serious biases because analyses of covariance are founded on the assumption of low interdependence between the covariate and the dependent variable (Owen and Froman, 1998).

Given contentious results regarding the interactions between bilingualism and SES, it is important to consider the context in which prior studies of bilingual EF were conducted. The majority of research examining the impact of bilingualism on cognitive control mechanisms has been conducted with bilingual participants who are well educated (Morton and Harper, 2009) and have income levels that are equivalent to those of monolingual English-speaking citizens. For example, in many of Bialystok’s studies (see commentary Bialystok, 2009) and in Morton and Harper (2007) study, the bilingual samples came from middle-class families. However, in the United States, the demographic characteristics are more complex, such that monolingual children and bilingual children often occupy distinct socio-demographic niches. Monolingual English-speaking children in the US are likely to be Caucasian, and to occupy middle SES households (Lopez and Velasco, 2011). Conversely, the largest bilingual population in the US is of Hispanic background, and likely to be first or second-generation immigrants. There are 10.9 million Hispanic students enrolled in the US schools (Kohler and Lazarín, 2007), and 26.9% of Hispanic immigrants in the US live in poverty (Camarota, 2012). Therefore, examining the effects of bilingualism on cognitive function in the context of a low SES Hispanic population is important, as the findings would be more likely to generalize to a large number of children and would speak to the possibility that bilingualism may offset the negative influences of poverty on EF development. However, comparing bilingual children from this background to their monolingual counterparts while matching the groups on SES, ethnicity, immigrant status, etc., is unfeasible. Furthermore, such a comparison is ecologically misaligned with the current demographic trends in the United States, where the majority of monolingual children do *not* share socio-demographic characteristics with Hispanic bilingual children. Apart from the difficulties associated with matching the demographic characteristics across bilingual and monolingual samples in studies of executive function, the examination of bilingual effects on EF is complicated by the lack of understanding as to the precise mechanisms that link non-linguistic cognitive control skills with language experience.

### MEASURING LINGUISTIC AND NON-LINGUISTIC COGNITIVE CONTROL SKILLS

Studies examining the relationship between linguistic and non-linguistic cognitive control skills are rare. In one recent study, Alario et al. (2012) examined the relationship between monolingual children’s performance on language tasks (picture naming and lexical decision) and their performance on non-verbal tasks (Simon and hue discrimination). Their analyses revealed null results in that there was no relationship between the linguistic and the non-linguistic tasks, indicating domain-specific cognitive control skills in monolinguals. However, studies of cross-domain relationships in bilingual populations have indicated possible linkages between linguistic and non-linguistic cognitive control mechanisms (Blumenfeld and Marian, 2011; Kaushanskaya et al., 2014).

Studies examining the relationship between linguistic and non-linguistic cognitive control skills in bilingual populations have mainly focused on code-switching and task-switching performance (e.g., Festman et al., 2010; Prior and Gollan, 2011; Weissberger et al., 2012). For example, Festman et al. (2010) found that those bilinguals who performed better on a picture naming task, a measure of linguistic control, also performed better on a measure of non-linguistic inhibitory control. Furthermore, a few studies that have compared the strength of relationships between domains in bilingual vs. monolingual speakers, have generally observed stronger links between cognitive and linguistic performance in bilinguals. For example, Blumenfeld and Marian (2011) examined the relationship between performance on an auditory processing task requiring resolution of phonological conflict and performance on a non-verbal Stroop task in bilingual vs. monolingual adults, and found a strong relationship between the linguistic and the non-linguistic tasks in their bilingual sample, but not in their monolingual sample. Similarly, in a recent study, Kaushanskaya et al. (2014) found strong relationships between verbal working memory and task-shifting performance in a sample of school-age children enrolled in Spanish–English dual-immersion programs, but not in a sample of monolingual school-age children attending English-only programs.

Thus, only a handful of studies examined the relationship between linguistic and non-linguistic skills in bilinguals, and these diverged greatly in tasks, populations, and methodologies. Furthermore, the existence of such linkages in the monolingual populations has been inconsistently observed (Alario et al., 2012), and the explanation of such linkages (or lack thereof) has been largely conjectural. For example, Blumenfeld and Marian (2011) accounted for the stronger link between conflict resolution on an auditory comprehension task and cognitive control in bilingual vs. monolingual adults by suggesting that resolution of competition during language processing and resolution of conflict during cognitive processing tap into the same mechanism in bilinguals but not in monolinguals. They attribute this to bilinguals’ greater need to resolve linguistic conflict. Conversely, Alario et al. (2012) attributed the null findings with regard to the relationship between verbal processing and cognitive control in monolingual participants to lower variability in the monolingual population with regard to both of these domains than in the bilingual population. Therefore, the main objective of the current study was to test the relationship between control skills in the linguistic and non-linguistic domain in monolingual and in bilingual children. The secondary goal was to explore the contribution of SES to bilingual children’s performance on the linguistic and the non-linguistic cognitive control tasks.

### THE CURRENT STUDY

The starting point for the present study was the observation that it is unfeasible, in the context of the United States, to compare a demographically representative sample of Spanish and English speaking bilingual children to a matched sample of English-speaking monolingual peers. That is, although it is possible to locate monolingual children from low SES backgrounds and bilingual children from middle SES backgrounds, these children would still mismatch on one or more of other characteristics that can influence EF abilities (such as immigrant status, family structure, ethnicity, etc.). Therefore, rather than taking the traditional comparative approach and searching for bilingual advantages or disadvantages while attempting a match in SES across the two in language groups, the present study focused on examining the relationship between linguistic and non-linguistic cognitive control abilities in a sample of monolingual children from middle SES backgrounds and in bilingual children from low SES backgrounds. In this way, we aimed to delineate the possible mechanisms that may give rise to cognitive advantages associated with bilingualism. Study 1 was designed to establish whether or not a relationship exists between linguistic and non-linguistic cognitive control skills in a sample of typically developing monolingual English-speaking children from middle SES backgrounds (the group traditionally studied by research focusing on EF abilities). Because of the sparseness of available research *directly* assessing the relationship between linguistic and non-linguistic cognitive control, such an approach is a necessary starting point for any work attempting to link language experience to cognitive control mechanisms. Study 2 was designed to explore this relationship in a sample of Spanish–English bilingual children from low SES backgrounds.

In order to examine linguistic cognitive control skills, a grammaticality judgment task similar to one developed by Bialystok (1986) and used by Lum and Bavin (2007) was designed. The task was designed to manipulate both grammar and semantics, such that children were required to only respond to the grammaticality of the sentences and to ignore their meaning. Therefore, this task is ideally suited to the purposes of this study because of the documented increased need to inhibit irrelevant semantic information in sentences that were grammatical but meaningless and in sentences that were ungrammatical but meaningful (Bialystok, 1986; Lum and Bavin, 2007). A flanker task was used to measure children’s non-linguistic inhibitory control. The flanker task required individuals to indicate the direction of a central arrow while ignoring the direction of flanking arrows, and has been used widely as a measure of non-linguistic inhibitory control in prior studies (e.g., Bialystok et al., 2004, 2008; Costa et al., 2008).

Children’s performance on the flanker was used to split the children into two inhibitory-control groups – the good inhibitors and the poor inhibitors. This grouping served as the independent variable, and performance on the GJ task served as the dependent variable. This approach enabled us to examine the relationship between children’s inhibitory control in the non-linguistic and linguistic domains. In Study 1, we examined the relationship between linguistic cognitive control and non-linguistic cognitive control in monolingual English-speaking children from middle SES backgrounds, representing the majority of the monolingual US population. In Study 2, we examined this relationship in Hispanic bilingual children from immigrant families and low SES backgrounds, representing the majority of the bilingual US population. We broadly hypothesized that if linguistic and non-linguistic cognitive control skills are mechanistically linked, then good inhibitors (identified based on their flanker performance) would outperform poor inhibitors on the grammaticality judgment task, especially in conditions where children were required to inhibit incongruent semantic information. Given that prior studies have indicated that both SES and bilingual status can influence cognitive-control performance, we also anticipated different patterns of relationships in monolingual children (Study 1) and bilingual children (Study 2).

## STUDY 1 METHOD

### PARTICIPANTS

Forty-six (30 boys) typically developing (TD) monolingual, English-speaking children (*M*_age_ = 8.50, SD = 0.78) participated in Study 1. Children’s primary caregivers completed a questionnaire regarding the children’s current medical status, socioeconomic status, medical history, and language development history. Children were retained for the analyses if they did not have disorders such as language impairment, developmental disabilities, or emotional or other psychological disorders. In order to rule out attentional deficits in the sample, the parents completed the *Conners 3rd Edition* (Conners, 2008), a questionnaire used to identify Attention Deficit Hyperactivity Disorder (ADHD). Any child who obtained a T-score higher than 60 on the Conners Inattention Scale and on the Conners Hyperactivity/Impulsivity Scale was excluded from the analyses. In addition, all participants passed a bilateral pure tone hearing screening at 25 dB.

In studies of language acquisition, SES is most often indexed by maternal level of education (Ensminger and Fothergill, 2003). In the current study, children’s SES was reported by primary caregivers in the background questionnaire. Primary caregivers indicated their level of education on a scale ranging from 1, Less than High School to 8, Professional/Doctoral Degree. The average SES for the sample was 6.26 (4-year college degree; SD = 1.10). The majority of the sample had either a 4-year college degree or a master’s degree. Of the total sample, four primary caregivers had a professional degree. This indicated that, on average, the sample came from middle to upper class SES backgrounds.

### PROCEDURE

#### Grammaticality judgment task

To measure children’s linguistic cognitive control skills, a grammaticality judgment task based on the task developed by Bialystok (1986) was used. Children heard four types of sentences: (1) grammatical and meaningful sentences (GM: *Dora is sliding down the red slide*); (2) ungrammatical and meaningful sentences (gM: *Yesterday, the baby cry all night long*); (3) grammatical and meaningless sentences (Gm: *The pillow talks to itself every night*); and (4) ungrammatical and meaningless sentences (gm: *The car is sit on the house*). Each sentence consisted of early acquired syntactic structures targeting knowledge of three grammatical rules (past tense, present progressive, and third person singular). Each sentence was 8 (±1) words in length.

First, children heard 24 practice sentences (six per each condition) to ensure understanding of the task. Children were instructed to attend to the “Word Rule,” in which they were required to make sure that there were no mistakes in any of the words and that there was nothing missing in each sentence. They were instructed *not* to attend to the “Meaning Rule,” that is, the whole idea of the sentence. If a child responded incorrectly during the practice trials, the experimenter provided explanatory feedback regarding whether or not that sentence followed the “Word Rule.” Following the practice trials, children heard a new set of 48 sentences (12 per condition), presented in a different randomized order to each child; children did not receive visual or verbal feedback on their performance. Children were instructed to respond as quickly as possible, and were allowed to respond at any time post sentence onset. As a result, although both accuracy and RT data were collected, only accuracy data were interpretable and analyzable.

#### Flanker task

To measure children’s non-linguistic inhibitory control, a flanker task (Eriksen and Eriksen, 1974) containing congruent and incongruent trials was used. The flanker task required the children to respond, as quickly as possible, to the direction of a middle arrow, which was flanked by two distracter arrows on both sides. On the congruent trials, the target arrow and the flanker arrows faced in the same direction. On the incongruent trials, flanker arrows were associated with a competing response (facing the opposite direction from the target arrow). Participants completed 10 practice trials prior to the experimental task in order to ensure understanding of the rules. Participants completed a total of 80 experimental trials (40 congruent and 40 incongruent), presented in a different randomized order to each child. Both accuracy (proportion correct) and RT data were collected. The RTs were recorded from the time that the children saw the display until they pressed a key in response. RTs that were above or below 2 SDs from a child’s mean were eliminated, resulting in the total loss of 4.19% of the RT data.

#### Standardized measures

Each child completed standardized measures of expressive and receptive language and non-verbal intelligence. A comprehensive language assessment, the *Clinical Evaluation of Language Fundamentals-Fourth Edition* (CELF-4; Semel et al., 2003), was used to evaluate each participant’s expressive (*M* = 115.15, SD = 12.36) and receptive (*M* = 112.76, SD = 12.12) language. To evaluate children’s nonverbal intelligence (*M* = 112.17, SD = 17.35), the *Visual Matrices* subtest of the *Kaufman Brief Intelligence Test* (KBIT-2; Kaufman and Kaufman, 2004) was used. These data confirmed that the children’s language abilities and non-verbal intelligence were within normal limits.

### DATA ANALYSES

The data were analyzed in three phases. First, children’s performance on the GJ task and the flanker task was examined in order to confirm that the two tasks were capturing the variables of interest (namely, increased difficulty with processing incongruent semantic information on the GJ task, and increased difficulty with processing conflicting visual information on the flanker task). These analyses were performed on the total sample. Second, children were split into two groups based on their performance on the flanker task, and the effect of this independent variable on GJ performance was examined. Lastly, bivariate correlations and multiple regressions were performed to examine whether there was a relationship between SES and children’s performance on the GJ and their flanker inhibition index, and whether SES and non-linguistic inhibitory control contributed independently to linguistic inhibitory control performance.

## RESULTS

### PERFORMANCE OF THE SAMPLE AS A WHOLE

#### Grammaticality judgment results

Accuracy data were analyzed using a 2 × 2 repeated-measures ANOVA with grammar and semantics as the independent variables. The ANOVA revealed a significant main effect of grammaticality, [*F*(1,44) = 23.28, *p* < 0.001] and a significant interaction between grammaticality and semantics, [*F*(1,44) = 12.65, *p* < 0.05]. The main effect of semantics was not statistically significant, [*F*(1,44) = 0.10, *p* = 0.76]. The significant two-way interaction was followed-up with two-tailed related-samples *t-*tests contrasting grammatical sentences that were congruent versus incongruent as well as ungrammatical sentences that were congruent versus incongruent. For the grammatical sentences (GM and Gm), children showed significant interference effects in that they were more accurate on the sentences in which grammar and semantics aligned (GM; *M* = 0.95, SD = 0.08) versus the sentences in which grammar and semantics did not align (Gm; *M* = 0.92, SD = 0.15), [*t*(44) = 2.31, *p* = 0.026; Cohen’s *d* = 0.31]. Likewise, for the ungrammatical sentences (gm and gM), children showed significant interference effects in that they were more accurate on the sentences in which grammar and semantics aligned (gm; *M* = 0.88, SD = 0.10) versus the sentences in which grammar and semantics did not align (gM; *M* = 0.84, SD = 0.08), [*t*(44) = 2.67, *p* = 0.011; *d* = 0.48].

#### Flanker results

Related-samples *t*-tests were performed to analyze the accuracy data on the flanker task and revealed that children were more accurate on the congruent trials (*M* = 0.90, SD = 0.12) compared to the incongruent trials (*M* = 0.86, SD = 0.14), [*t*(45) = 3.89, *p*< 0.001; *d* = 0.33]. Similarly, children were quicker to respond to the congruent trials (*M* = 644.04, SD = 87.36) versus the incongruent trials (*M* = 658.81, SD = 93.00), [*t*(45) = –2.87, *p* < 0.01; *d* = –0.16].

### ANALYSES LINKING GJ AND FLANKER PERFORMANCE

The flanker accuracy data were used to derive a flanker inhibition index because the use of accuracy data as both a predictor and an outcome variable served to maintain consistency across the two tasks. The flanker inhibition index was calculated by subtracting accuracy scores on the incongruent trials from the accuracy scores on the congruent trials for each child. Children with scores closer to zero would therefore have better inhibitory control skills than children with positive difference scores between congruent and incongruent trials. A median-split was performed on the flanker inhibition index data (*M* = 0.03). The median split resulted in two groups of children: good inhibitors (*n* = 19) and poor inhibitors (*n* = 26). Children with good inhibitory skills were those whose inhibition index was below the median (i.e., below 0.03, Range: –0.07 to 0.28) and children with poor inhibitory skills were those whose inhibition index was at or above the median (i.e., 0.03 and above). The two groups were compared to each other on demographic variables and language/cognitive measures in order to check for the possibility that the median-split procedure yielded uneven groups with regards to these background variables. Independent-samples *t*-tests with inhibitory-index group as the between-subjects independent variable revealed that the two groups significantly differed only on the flanker inhibition score [*t* (44) = –7.30, *p*< 0.001]. The two groups did not differ on any demographic or language/cognitive characteristics (all *p* values >0.1; see **Table 1**).

**Table 1:** Study 1: Monolingual background information for the good inhibitors and the poor inhibitors.

	Good inhibitors	Poor inhibitors	*t-*test
N	19	26	
Flanker inhibition index	–0.02 (0.02)	0.09 (0.01)	*t*(44) = –7.30*
Age (years)	8.73 (0.85)	8.34 (0.69)	*t*(44) = 1.68
SES	6.21 (1.23)	6.29 (1.03)	*t*(44) = –0.26
Non-verbal IQ	114.37 (15.09)	110.63 (18.90)	*t*(44) = 0.72
Receptive language	114.15 (12.98)	111.15 (11.44)	*t*(44) = 0.44
Expressive language	118.58 (13.17)	112.74 (11.40)	*t*(44) = 0.89

*Significant at *p* < 0.001.

In order to examine the relationship between inhibitory-control skills and GJ performance, the four grammaticality judgment conditions were collapsed into two conditions, congruent (GM and gm) and incongruent (Gm and gM), in order to increase statistical power. Independent-samples *t-*tests were used to examine differences in performance between the good inhibitors and the poor inhibitors on the two grammaticality judgment conditions. There was a trend for the good inhibitors (*M* = 0.94, SD = 0.05) to outperform the poor inhibitors (*M* = 0.91, SD = 0.09) on the *congruent GJ* condition, but this difference was not statistically significant, [*t*(43) = 1.27, *p* = 0.21; *d* = 0.40]. However, when accuracy data for the *incongruent GJ* condition were considered, the good inhibitors (*M* = 0.91, SD = 0.04) were found to significantly outperform the poor inhibitors (*M* = 0.85, SD = 0.11), [*t*(43) = 2.13, *p*< 0.05; *d* = 0.68].

### ASSESSING THE IMPACT OF SES

Correlation analyses revealed a significant relationship between GJ incongruent accuracy data and SES (*r incongruent* = 0.39, *p*< 0.05). However, lack of relationships was observed between SES and the flanker inhibition index (*r* = –0.07, *p* = 0.64), and the GJ congruent accuracy data (*r congruent* = 0.11, *p* = 0.45).

Next, two separate regression models were built, one for the GJ congruent data and one for the GJ incongruent data. Children’s SES was entered as the initial predictor, followed by the flanker inhibition index. The GJ data served as the dependent variable. Together, SES and the flanker inhibition index explained 10.4% of the variance in the *congruent condition* [*R^2^* = 0.10, *F*(2,42) = 2.24, *p* = 0.10]. While SES did not significantly predict congruent GJ accuracy (β = 0.09, *p* = 0.57), the flanker inhibition index did (β = –0.30, *p* < 0.05). Together, SES and flanker inhibition index explained 17.5% of the variance in the* incongruent condition* [*R^2^* = 0.17, *F*(2,42) = 4.46, *p*< 0.05]. The flanker inhibition index marginally predicted incongruent GJ accuracy (β = –0.24, *p* = 0.09), while SES predicted it significantly (β = 0.32, *p* < 0.05).

## STUDY 1 DISCUSSION

Study 1 was designed to examine the relationship between linguistic and non-linguistic cognitive control skills in typically developing English-speaking monolingual children from middle-class backgrounds. These children represent the majority of monolingual populations in the US. We found that our sample of monolingual English-speaking children experienced significant interference from incongruent semantic information when engaging in a grammaticality judgment task. In line with previous studies that have observed a similar result (Bialystok, 1986; Lum and Bavin, 2007), we interpret these findings to suggest that children were required to inhibit the task-irrelevant semantic information in order to attend to the task-relevant grammatical information. The novel finding in the current study is that children with better inhibitory control skills (as captured by the non-linguistic flanker task) were better able to inhibit irrelevant semantic information when processing grammatical information. The finding that the differences between good and poor inhibitors held only for the incongruent but not the congruent sentences suggests that the link between non-linguistic control and GJ performance may be specific to the linguistic tasks that involve inhibition.

Based on results in Study 1, it can be concluded that monolingual children may recruit domain-general inhibitory control mechanisms during demanding linguistic processing tasks. Importantly, such demands for heightened inhibitory control during linguistic processing are likely to characterize not just performance on this one very specific grammaticality judgment task but instead the vast majority of interactions that children may be involved in on a day-to-day basis. This is because most processing of language occurs in noisy environments where a variety of competing linguistic (and non-linguistic) information must be ignored in order to zero in on the relevant linguistic information. Therefore, the finding that typically-developing monolingual children who have poorer domain-general attention skills also have poorer ability to process linguistic information is significant in that it suggests that even within the gamut of normal language and attention skills, lower attention may constrain linguistic abilities.

The question remains whether the same link between domain-general inhibitory control mechanisms and linguistic inhibitory control mechanisms would exist for bilingual speakers. Such a relationship between linguistic and non-linguistic cognitive control skills has been implied in the bilingualism literature, with bilingual advantages in non-linguistic cognitive control attributed to the need to exercise cognitive control during linguistic processing (Bialystok, 2001). However, there is limited evidence for a direct link between linguistic and non-linguistic cognitive control mechanisms in bilinguals. Study 2 was designed to explore the relationship between linguistic and non-linguistic cognitive control skills in an ecologically representative sample of low SES bilingual children residing in the US.

## STUDY 2 METHOD

Thirty-nine (15 boys) typically developing (TD) bilingual, Spanish-English-speaking children (*M_age_* = 8.25, SD = 0.97) participated in Study 2. The bilingual children’s primary caregivers completed a questionnaire regarding the children’s current medical status, socioeconomic status, medical history, and language development history. The same exclusionary criteria as the ones applied to the monolingual sample in Study 1 were used in Study 2. Any child who obtained a T-score higher than 60 on the Conners Inattention Scale and on the Conners Hyperactivity/Impulsivity Scale of the *Conners 3rd Edition* (Conners, 2008) was eliminated from analyses. All participants had hearing within normal limits, which was confirmed by a bilateral pure tone hearing screening at 25 dB.

The majority of the children were of Latino background (62%), 10% were multiracial, and 28% were identified by their primary caregivers as Caucasian. As a group, the children began producing two-word phrases in English (*M* = 31.32, SD = 20.77) and in Spanish (*M* = 37.37, SD = 21.83) around the same time. For the majority of children, the caregivers reported Spanish as the native language (49%). For 33% of the participants, the caregivers identified English as the native language, and for 18% of the participants, the caregivers reported acquisition of both English and Spanish from birth. Most children attended English-speaking schools (67%), with the rest of the children (33%) attending Spanish-English dual immersion programs. Per parent report, the majority of the children preferred to speak English (69%), while 19% preferred English and Spanish equally, and 12% preferred to speak Spanish.

Children’s SES was reported by primary caregivers on the scale ranging from 1 (Less than High School) to 8 (Professional/Doctoral Degree). The average SES for the bilingual sample was 4.29 (some college; SD = 2.31). Specifically, 22.2% of the primary caregivers had less than a high school education; 14.6% had some high school education; 25.9% had a high school or a GED equivalent education; 11.1% had some college; 7.4 % had a 2-year college degree; 14.8% had a 4-year college degree; and 3.7 % had a master’s degree.

### PROCEDURE

The bilingual children in Study 2 completed the same experimental tasks and standardized measures as the monolingual children in Study 1. For the flanker task, 2.63 % of the RT data were eliminated from the analyses as outliers. The CELF-4 was used to evaluate each bilingual participant’s English expressive (*M* = 97.66, SD = 16.47) and English receptive (*M* = 92.43, SD = 20.16) language. The *Visual Matrices* of the KBIT-2 revealed average non-verbal intelligence skills (*M* = 103.92, SD = 17.89). All bilingual children were also administered Spanish-language measures. Specifically, the *Clinical Evaluation of Language Fundamentals-Fourth Edition, Spanish* (CELF-4 Spanish; Wiig et al., 2006) was used to measure each participant’s Spanish expressive (*M* = 86.36, SD = 9.69) and Spanish receptive (*M* = 97.07, SD = 12.39) language skills. The data confirmed that the children’s Spanish and English language abilities and non-verbal intelligence were within normal limits.

## RESULTS

### PERFORMANCE OF THE SAMPLE AS A WHOLE

#### Grammaticality judgment results

Accuracy data were analyzed using a 2 × 2 repeated-measures ANOVA with grammar and semantics as the independent variables. The ANOVA revealed a significant main effect for grammaticality, [*F*(1,38) = 24.13, *p* < 0.001] and a significant interaction between grammaticality and semantics, [*F*(1,38) = 7.19, *p* < 0.05]. The main effect of semantics was not statistically significant, [*F*(1,38) = 0.05 *p* = 0.82]. The significant two-way interaction was followed-up with two-tailed related-samples *t-*tests contrasting grammatical sentences that were congruent versus incongruent as well as ungrammatical sentences that were congruent versus incongruent. For the grammatical sentences (GM and Gm), bilingual children showed significant interference effects in that they were more accurate on the sentences in which grammatical and semantic information aligned (GM; *M* = 0.87, SD = 0.17) versus the sentences in which grammatical and semantic information did not align (Gm; *M* = 0.79, SD = 0.25), [*t*(38) = –2.19, *p* = 0.03; *d* = 0.37]. A similar pattern was observed for the ungrammatical sentences (gm and gM), where children showed significant interference effects and were more accurate on the sentences in which grammatical and semantic information aligned (gm; *M* = 0.68, SD = 0.21) versus the sentences in which grammatical and semantic information did not align (gM; *M* = 0.61, SD = 0.24), [*t*(38) = 2.43, *p* = 0.02; *d* = 0.31].

#### Flanker results

Related-samples *t*-tests were performed to analyze the accuracy data on the flanker task and revealed that bilingual children were more accurate on the congruent trials (*M* = 0.85, SD = 0.12) compared to the incongruent trials (*M* = 0.76, SD = 0.19), [*t*(36) = 3.83, *p*< 0.001; *d* = 0.57]. However, no significant differences were observed in reaction time data in that children responded equally quickly to the congruent trials (*M* = 675.74, SD = 87.32) and the incongruent trials (*M* = 685.54, SD = 86.29), [*t*(36) = –1.56, *p* = 0.13; *d* = –0.11].

### ANALYSES LINKING GJ AND FLANKER PERFORMANCE

The median split of the flanker accuracy data resulted in a group of 17 good inhibitors and a group of 21 poor inhibitors. One child’s data were not included due to missing flanker data. The median inhibition index was 0.05 (Range: –0.15 to 0.45). Therefore, children with good inhibitory skills were those whose inhibition index was below 0.05 and children with poor inhibitory skills were those whose inhibition index was at or above 0.05. The two groups significantly differed only on the flanker inhibition score [*t*(36) = –6.77, *p*< 0.01]. They did not differ on any demographic characteristics (all *p* values > 0.1; see **Table 2**).

**Table 2:** Study 2: Bilingual background information for the good inhibitors and the poor inhibitors.

	Good inhibitors	Poor inhibitors	*t-*test
N	17	21	
Flanker inhibition index	–0.03 (0.05)	0.18 (0.12)	*t*(35) = –6.77*
Age (years)	8.06 (0.89)	8.45 (1.01)	*t*(36) = –1.26
SES	4.88 (2.36)	4.00 (2.19)	*t*(36) = 1.16
Non-verbal IQ	108.69 (18.07)	100.10 (17.25)	*t*(36) = 1.45
English receptive language	100.38 (18.50)	95.37 (14.66)	*t*(35) = 0.89
English expressive language	92.63 (21.95)	92.26 (19.14)	*t*(35) = 0.05
Spanish receptive language	99.00 (13.66)	95.38 (11.35)	*t*(35) = 0.79
Spanish expressive language	84.00 (10.18)	88.06 (9.26)	*t*(35) = –1.08

*Significant at *p* < 0.001.

As in Study 1, the four grammaticality judgment conditions were collapsed into two conditions, congruent (GM and gm) and incongruent (Gm and gM), in order to increase statistical power and to examine the relationship between inhibitory-control skills and GJ performance. Independent-samples *t-*tests yielded no significant results. Specifically, the analyses showed that the good inhibitors (*M* = 0.82, SD = 0.12) and the poor inhibitors (*M* = 0.74, SD = 0.17) performed similarly on the *congruent GJ* condition, [*t*(36) = 1.56, *p* = 0.07; *d* = 0.54]. Likewise, the data for the *incongruent GJ* condition did not yield significant results; the good inhibitors (*M* = 0.70, SD = 0.23) and the poor inhibitors (*M* = 0.71, SD = 0.18), [*t*(36) = –0.04, *p* = 0.39; *d* = –0.05] performed similarly.

### ASSESSING THE IMPACT OF SES

Correlation analyses revealed a significant relationship between SES and GJ data (*r congruent* = 0.43, *p*< 0.05; *r incongruent* = 0.39, *p*< 0.05). However, a lack of a relationship was observed between SES and the flanker inhibition index (*r* = –0.02, *p* = 0.93).

As in Study 1, two separate regression models were built to assess the impact of SES and the flanker inhibition index on the GJ performance. Together, SES and the flanker inhibition index explained 16.4% of the variance in the *congruent condition* [*R^2^* = 0.16, *F*(2,33) = 3.23, *p* = 0.05]. SES significantly predicted congruent GJ accuracy (β = 0.38, *p* < 0.05), but the flanker inhibition index did not (β = –0.12, *p* = 0.44). Together, SES and the flanker inhibition index explained 13.7% of the variance in the *incongruent condition* [*R^2^* = 0.14, *F*(2,33) = 2.61, *p* = 0.09]. SES significantly predicted incongruent GJ accuracy (β = 0.35, *p* < 0.05), while the flanker inhibition index did not (β = 0.10, *p* = 0.53).

## STUDY 2 DISCUSSION

Study 2 was designed to examine whether a relationship between non-linguistic and linguistic cognitive control skills would be observed in a sample of socio-demographically representative bilingual children in the US. Grammaticality judgment data revealed that, just like the monolingual group in Study 1, and similar to other studies that have used the GJ task to index linguistic cognitive control (Bialystok, 1986; Lum and Bavin, 2007), the bilingual group in Study 2 experienced significant interference from incongruent semantic information when engaging in a grammaticality judgment task. Consistent with the classic interpretation of these interference effects (Bialystok, 1986; Lum and Bavin, 2007), we ascribe the differences observed in GJ performance between congruent and incongruent sentences in the bilingual group to the need to suppress incongruent semantic information while responding to the grammaticality of the sentence. However, in contrast to Study 1, in Study 2, when bilingual children were divided into two groups (*good inhibitors* and *poor inhibitors*) based on their performance on the non-linguistic cognitive control task, no differences were observed between the two groups on their performance on the grammaticality judgment task.

Based on prior research regarding bilingual advantages in the realm of EF (e.g., Kroll and Bialystok, 2013), we expected bilingual children to show links between linguistic and non-linguistic cognitive control that would be at least as strong as those observed for the monolingual children in Study 1. Instead, we found that Spanish-English bilingual children from lower SES backgrounds experienced interference from competing semantic information when processing sentences in English, but did not appear to recruit domain-general cognitive control skills to resolve this interference. The lack of significant differences between the good inhibitors and the poor inhibitors in Study 2 may be attributed to bilinguals’ lower English language skills. That is, perhaps English language knowledge in this group was too low to engender activation of semantic information during grammatical processing, thus nullifying the need to apply inhibition-based mechanisms during the processing of the incongruent sentences. However, this explanation is unlikely, due to two considerations. First, the bilingual group as a whole performed within the average range on standardized English language measures when compared to monolingual test norms. Second, we observed significant differences between congruent and incongruent conditions on the GJ task in the accuracy data in Study 2. Therefore, the bilingual children we tested had sufficient knowledge of English to experience interference from semantics during grammatical processing. We propose that the lack of a relationship between linguistic and non-linguistic inhibitory control in Study 2 reflects a true segregation of the two mechanisms in this bilingual sample.

The findings of Study 2 may be specific to the group of bilingual children tested here, and indeed it may be that in a group of bilingual children from a different socio-demographic and linguistic background, a different pattern of results would be observed. Bilingual advantages may be present for some bilingual populations but may not be present in others, and language history together with SES are just some aspects that will vary when sampling across bilingual populations. The fact that Study 2 did not find a relationship between linguistic and non-linguistic cognitive control skills in a sample of bilingual children in the U.S. from low SES suggests that it is important to consider environmental factors that can modulate the relationship between linguistic and non-linguistic cognitive control. It may be beneficial to move beyond the question of whether or not there are bilingual cognitive advantages and instead consider the role of environmental factors (including bilingualism) in the development of language and cognitive skills, in a more continuous and demographically representative manner. Although the present study cannot dissociate between SES and bilingualism in their influences of cognitive control, it does identify plausible directions for future investigation of demographic variables that may impact the development of both non-linguistic and linguistic cognitive skills. In the General Discussion, we outline the mechanisms that may underlie the significant relationships between non-linguistic and linguistic cognitive control skills in monolinguals, and propose a number of hypotheses for the lack of such a relationship in bilinguals.

## GENERAL DISCUSSION

In two studies, we examined the relationship between linguistic and non-linguistic cognitive control skills in two populations, monolingual English-speaking children and bilingual Spanish-English speaking children. The present paper is the first to examine *direct* links between linguistic and non-linguistic cognitive control skills in monolingual children and in bilingual children. The two studies demonstrated that typically developing monolingual English-speaking children from middle SES backgrounds as well as bilingual English-Spanish speaking children from low SES backgrounds found it difficult to process grammatical information in the context of conflicting semantic information. Further, monolingual children with better non-linguistic inhibitory control skills were better able to manage this linguistic conflict, suggesting recruitment of domain-general cognitive-control skills for linguistic processing. However, bilingual children did not appear to recruit non-linguistic cognitive control skills during linguistic processing, suggesting that bilinguals, unlike monolinguals, may rely on local inhibitory control skills only (i.e., only linguistic inhibition) for resolving conflict in the linguistic domain.

Studies that attempt to directly link non-linguistic cognitive control and linguistic cognitive control are rare in both monolinguals and bilinguals. The only study to directly examine this relationship in monolinguals reported null results (Alario et al., 2012). A possible explanation for the different results in Alario et al. (2012) study and our Study 1 may be that the present study used a more complex linguistic task than the picture naming and the lexical decision tasks used by Alario et al. (2012). A few studies that have examined the strength of relationships between a linguistic processing task and a non-linguistic executive-function task in bilingual vs. monolingual speakers (Blumenfeld and Marian, 2011; Prior and Gollan, 2011; Kaushanskaya et al., 2014) reported, in general, stronger links between cognitive and linguistic performance in bilingual populations. However, our findings suggest the opposite. While Study 1 provided evidence that monolingual children recruit non-linguistic inhibitory control skills when processing complex (incongruent) syntactic information, Study 2 provided evidence that bilingual children do not. Two parameters associated with Study 2 may be the causal factors behind the different patterns of results between our work and prior studies attempting to link non-linguistic and linguistic processing in bilinguals (Blumenfeld and Marian, 2011; Kaushanskaya et al., 2014). First, the recruitment of bilingual children from low SES backgrounds for Study 2 not only made it impossible for us to compare them directly to the monolingual children from mid-SES backgrounds for Study 1, but also made it possible for SES to exert an influence on both linguistic and non-linguistic performance. Second, for the majority of the bilingual children in our study, English was the second language (L2), whereas previous studies that have identified stronger linkages between linguistic and non-linguistic tasks in bilinguals have tested bilinguals who performed the linguistic task in their native language.

With regard to SES being the mediating factor behind the different patterns of results observed between Study 2 and Study 1, both SES and bilingualism likely bear relationships to linguistic development and the development of cognitive skills in children. For instance, it is well documented that lower SES is associated with reduced gains in children’s cognitive (Noble et al., 2005) and linguistic development (Hart and Risley, 1995). Similarly, bilingualism has an impact on language processing in that bilinguals are often reported to be slower than monolinguals on language comprehension (e.g., Kroll et al., 2008) and production tasks (e.g., Gollan et al., 2005). Therefore, it may be that the combination of low SES and an unstable language processing system instantiated in our bilingual children in Study 2 imposed fundamentally different processing demands on the GJ task for the bilingual children.

Evidence for different mechanisms that may underlie bilinguals’ vs. monolinguals’ performance on the GJ task comes from correlation and regression analyses where SES was examined in relation to children’s performance on the GJ task. For monolingual children in Study 1, SES bore a limited relationship to GJ performance, whereas for bilingual children in Study 2, SES was strongly linked to GJ performance. The finding that SES has a stronger predictive power over second-language performance vs. native-language performance is consistent with prior studies of SES and linguistic development in bilinguals (e.g., Quiroz et al., 2010; Hammer et al., 2012; Buac et al., 2014). For instance, in our prior work (Buac et al., 2014), we showed that SES was more strongly related to bilingual children’s vocabulary skills in their second language (English) than in their native language (Spanish). In our current study, the pattern of results indicates that bilingual children’s performance on the English GJ task was likely affected by both children’s bilingual status as well as by their SES. This may have diluted the mechanistic link between the inhibitory control bilingual children exercised during the linguistic task, and the inhibitory control they exercised during the non-linguistic task.

Another interpretation of the different patterns of results observed for the bilingual children in Study 2 is to ascribe these to second-language vs. native-language processing demands. That is, the lack of relationships between non-linguistic and linguistic cognitive control in bilingual children can be interpreted to suggest that linguistic processing in the second language may be less linked to global cognitive control. The GJ task used in the present study required children to suppress semantic information in English. For the majority of bilingual children, this meant that they were required to process and ignore semantic information in their relatively weaker second language. The lack of the relationship between GJ and flanker data for the bilingual group may indicate that activation of semantic information in the L2 during the GJ task may not have exceeded a critical threshold that would require active inhibition. This interpretation may be consistent with the Revised Hierarchical Model of L2 processing (Kroll and Stewart, 1994), which posits weak direct links between the L2 lexical system and the conceptual store during the early phases of L2 acquisition (but see Brysbaert and Duyck, 2010). If we consider the bilingual children in our study to be in relatively early phases of English acquisition, then it is possible that incongruent semantic information was activated strongly enough to interfere with grammatical processing, but not strongly enough to require top-down domain-general inhibition mechanisms to come on line to resolve the interference.

Yet another possibility behind the different patterns of results in Study 1 vs. Study 2 may be that the flanker task captured different capabilities in the monolingual versus the bilingual group. The monolingual children in Study 1 were both less accurate and slower on the incongruent flanker trials – a pattern of results typically interpreted to suggest difficulty with resolving perceptual conflict. However, the bilingual children in Study 2 were impacted by the incongruency of the arrows only in the accuracy data, with RT data being comparable across the congruent and the incongruent conditions. In prior studies of bilingual executive function, such lack of RT differences between congruent and incongruent conditions was interpreted as successful inhibition of the incongruent spatial dimension, and thus an advantage on non-linguistic inhibitory control tasks (e.g., Costa et al., 2008, 2009). However, in our study, the presence of the incongruency effects in the accuracy data indicates that the bilingual children experienced significant interference from the incongruent spatial information on the flanker task, but that this interference did not pervade all aspects of performance. If our flanker task was unsuccessful in capturing non-linguistic inhibition in bilinguals, then it would be unlikely to predict linguistic inhibition in this group.

Finally, it is important to point out that the directionality of a relationship between non-linguistic cognitive control and linguistic cognitive control is not a forgone conclusion, and it is possible that linguistic skills contribute to non-linguistic cognitive control. That is, the ability to linguistically formalize the response rule may facilitate performance on a seemingly non-linguistic task such as the flanker. In previous work, just such a role of language in the development of the executive functions was proposed (Zelazo et al., 1996; Zelazo and Frye, 1998; Zelazo et al., 2003), with a number of studies demonstrating that labeling can improve performance on some classically non-linguistic executive-function measures (e.g., Kirkham et al., 2003; Müller et al., 2004). Such an interpretation may inform the results of Study 2, where the lack of the relationships between the GJ and the flanker data could be interpreted as bilingual children’s inability (or lack of need) to use their second language to formalize response rules while performing the flanker task. Studies that examine the directionality of the relationship between linguistic and non-linguistic cognitive control skills are crucial. In principle, the present experiments could have approached the question regarding the relationship between linguistic and non-linguistic cognitive control skills from the reverse hypothesis—that linguistic control skills may drive cognitive control skills. In fact, when we conducted the analyses that would test this hypothesis (by splitting children up based on their GJ scores and examining group differences on the Flanker task), we observed that in the monolingual group, children who performed better on the GJ task performed better on the incongruent, but not the congruent, condition of the Flanker task. However, in the bilingual group, there were no significant differences between children split on the basis of their GJ performance. Because of the cross-sectional nature of this study, analyses that test both directions of influence are redundant with each other. It is therefore prudent to state that the findings of the present work suggest that linguistic and non-linguistic cognitive control skills are more tightly bound in monolingual children than in bilingual children, without specifying the direction of the effects. Future work that takes both sides of this causal chain into account, and considers the development of linguistic and the non-linguistic cognitive control skills longitudinally in groups of children that span the continuum of linguistic ability (native speakers to second-language learners; typically developing children to children with language impairments) and SES backgrounds are necessary before we can state with certainty whether (and how) linguistic and cognitive mechanisms interact in development.

In general, it is evident that further research is necessary in order to substantiate the existence of links between linguistic and non-linguistic cognitive control mechanisms, and to examine the relative strength of these links across bilingual vs. monolingual populations. The ability to conduct such research is complicated by the findings that bilingualism may influence different components of cognitive control skills distinctly (see Hilchey and Klein, 2011 for a detailed review), that different aspects of bilingual experience may yield distinct influences on cognition (Blumenfeld and Marian, 2009; Barac and Bialystok, 2012), and that developmental stages interact with bilingualism in shaping the EF (Barac and Bialystok, 2012). We interpret our findings in Study 1 to suggest that linguistic and non-linguistic inhibitory control skills may mutually reinforce each other in monolingual children. We interpret our findings in Study 2 to suggest that linguistic inhibitory control skills exercised in the second language may arise independently from non-linguistic cognitive control skills used to resolve perceptual conflict.

Of course, all such interpretations of the different patterns of findings in Study 1 vs. Study 2 must be taken cautiously. The groups of children in the two studies represent common US demographic trends, with monolingual English-speaking children occupying middle SES households and bilingual Spanish-English speaking children occupying low SES households. However, the two samples likely differ not just in their SES, but also in their language abilities, ethnic and cultural identities, immigrant status, family structures, etc. Thus, it is difficult to attribute the different relationships between linguistic cognitive control and non-linguistic cognitive control within each group to any one individual factor that distinguishes them. The present study took only the first step towards identifying whether a relationship between linguistic and non-linguistic cognitive control skills holds in samples of monolingual and bilingual children that represent the widest segments of their respective populations.

## Conflict of Interest Statement

The authors declare that the research was conducted in the absence of any commercial or financial relationships that could be construed as a potential conflict of interest.
